# {2-[(2,6-Difluoro­phen­oxy)meth­yl]phen­yl}boronic acid

**DOI:** 10.1107/S1600536809035235

**Published:** 2009-09-05

**Authors:** Tomasz Klis, Janusz Serwatowski

**Affiliations:** a Physical Chemistry Department, Faculty of Chemistry, Warsaw University of Technology, Noakowskiego 3, 00-664 Warsaw, Poland

## Abstract

The planes of the two benzene rings in the mol­ecule of the title compound, C_13_H_11_BF_2_O_3_, form a dihedral angle of 76.06 (3)°; the C—O—C—C torsion angle characterizing the conformation of the central link of the mol­ecule is −79.20 (1)°. The dihydroxy­boron group is not coplanar with the benzene ring bonded to the B atom; one of the C—C—B—O torsion angles is 32.39 (2)°. One of the OH groups of the boronic acid fragment is engaged in an intra­molecular hydrogen bond, whereas the second OH group participates in inter­molecular hydrogen bonding, which leads to the formation of centrosymmetric dimers.

## Related literature

For applications of boronic acids and aryl-benzyl ethers, see: Bien *et al.* (1995 ▶); Dai *et al.* (2009 ▶); Miyaura & Suzuki (1995 ▶). For the structure of a related boronic acid, see: Serwatowski *et al.* (2006 ▶).
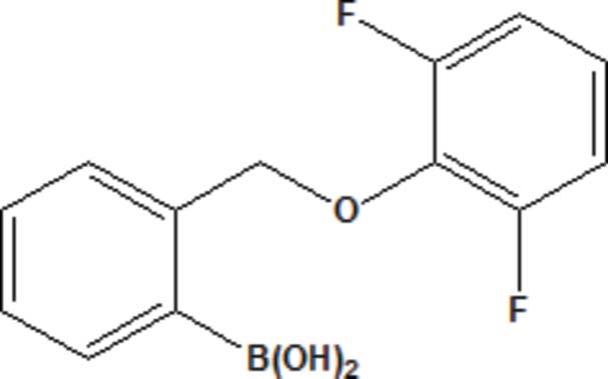


## Experimental

### 

#### Crystal data

C_13_H_11_BF_2_O_3_
*M* *_r_* = 264.03
Monoclinic, 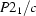

*a* = 7.6660 (7) Å
*b* = 14.2299 (13) Å
*c* = 11.3595 (13) Å
β = 101.146 (9)°
*V* = 1215.8 (2) Å^3^
*Z* = 4
Mo *K*α radiation
μ = 0.12 mm^−1^
*T* = 100 K
0.77 × 0.49 × 0.31 mm

#### Data collection

Oxford Diffraction KM-4-CCD diffractometer
Absorption correction: multi-scan (*CrysAlis RED*; Oxford Diffraction, 2005 ▶) *T*_min_ = 0.905, *T*_max_ = 0.964
16118 measured reflections
2969 independent reflections
2398 reflections with *I* > 2σ(*I*)
*R*_int_ = 0.014

#### Refinement

*R*[*F* ^2^ > 2σ(*F* ^2^)] = 0.030
*wR*(*F* ^2^) = 0.082
*S* = 1.09
2969 reflections
217 parameters
All H-atom parameters refined
Δρ_max_ = 0.32 e Å^−3^
Δρ_min_ = −0.20 e Å^−3^

### 

Data collection: *CrysAlis CCD* (Oxford Diffraction, 2005 ▶); cell refinement: *CrysAlis RED* (Oxford Diffraction, 2005 ▶); data reduction: *CrysAlis RED*; program(s) used to solve structure: *SHELXS97* (Sheldrick, 2008 ▶); program(s) used to refine structure: *SHELXL97* (Sheldrick, 2008 ▶); molecular graphics: *DIAMOND* (Brandenburg, 1999 ▶); software used to prepare material for publication: *SHELXTL* (Sheldrick, 2008 ▶).

## Supplementary Material

Crystal structure: contains datablocks I, global. DOI: 10.1107/S1600536809035235/ya2103sup1.cif

Structure factors: contains datablocks I. DOI: 10.1107/S1600536809035235/ya2103Isup2.hkl

Additional supplementary materials:  crystallographic information; 3D view; checkCIF report

## Figures and Tables

**Table 1:** Hydrogen-bond geometry (Å, °)

*D*—H⋯*A*	*D*—H	H⋯*A*	*D*⋯*A*	*D*—H⋯*A*
O1—H1*O*⋯O3	0.821 (16)	1.915 (16)	2.6926 (11)	157.7 (15)
O2—H2*O*⋯O1^i^	0.853 (17)	1.937 (17)	2.7889 (11)	176.9 (16)

Symmetry code: (i) 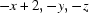.

**Fig. 1.**
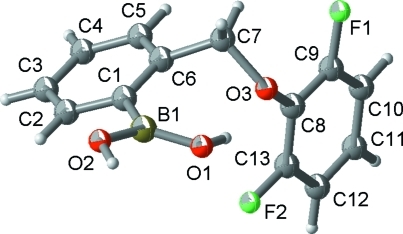
Molecular structure of (I) showing the atom labelling scheme. Displacement ellipsoids for non-H atoms are drawn at the 50% probability level; H atoms are shown as small circles of arbitrary radius.

**Fig. 2.**
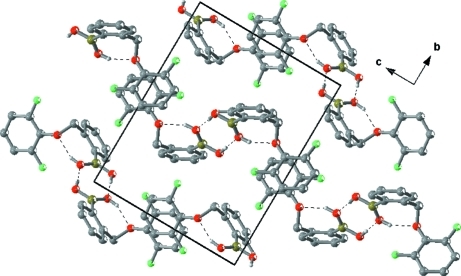
The crystal packing of (I) viewed along the a axis. H atoms not involved in hydrogen bonds (dashed lines) were omitted for clarity.

## supplementary crystallographic information

### Comment

The high synthetic utility of boronic acids (Bien *et al.*, 1995; Miyaura & Suzuki, 1995) boosts continuous progress in the preparation and characterization of these compounds. The title compound, C_13_H_11_BF_2_O_3_(I), is the first example of the arylboronic acid based on the aryl-benzyl ether core containing aryloxymethylene substituent. The structure of arylboronic acid with benzyloxy substituent has been published a few years ago (Serwatowski *et al.*, 2006). Aryl-benzyl ethers found recently their new application as human immunodeficiency virus-1 (HIV-1) inhibitors (Dai *et al.*, 2009).

The planes of two benzene rings in the molecule of (I) (Fig. 1) form the dihedral angle of 76.06 (3)° and the torsion angle C8—O3—C7—C6 characterizing the conformation of the central link of the molecule, is equal to -79.20 (1)°. The dihydroxyboron group is not coplanar with the benzene ring to which the B1 atom is attached: the C6—C1—B1—O1 torsion angle is equal to 32.39 (2)°.

The hydrogen atom bonded to O1 is involved in a relatively weak intramolecular O1—H1O···O3 bond. The hydrogen atom at the O2 atom paricipates in the intermolecular hydrogen bonding, which leads to the formation of centrosymmetric dimers (Table 1, Fig. 2).

### Experimental

The title compound was received from Aldrich; crystals suitable for X-ray study were grown from toluene.

### Refinement

Hydrogen atoms were located in the difference map and refined isotropically; C—H 0.946 (15)–0.978 (15) Å; O1—H1O 0.821 (16) and O1—H2O 0.853 (17) Å.

### Crystal data

**Table:**

C_13_H_11_BF_2_O_3_	*F*(000) = 544
*M**_r_* = 264.03	*D*_x_ = 1.442 Mg m^−^^3^
Monoclinic, *P*2_1_/*c*	Mo *K*α radiation, λ = 0.71073 Å
Hall symbol: -P 2ybc	Cell parameters from 7068 reflections
*a* = 7.6660 (7) Å	θ = 57.4–2.7°
*b* = 14.2299 (13) Å	µ = 0.12 mm^−^^1^
*c* = 11.3595 (13) Å	*T* = 100 K
β = 101.146 (9)°	Prismatic, colourless
*V* = 1215.8 (2) Å^3^	0.77 × 0.49 × 0.31 mm
*Z* = 4	

### Data collection

**Table:**

Oxford Diffraction KM-4-CCD diffractometer	2969 independent reflections
Radiation source: fine-focus sealed tube	2398 reflections with *I* > 2σ(*I*)
graphite	*R*_int_ = 0.014
Detector resolution: 8.6479 pixels mm^-1^	θ_max_ = 28.7°, θ_min_ = 2.7°
ω scans	*h* = −10→10
Absorption correction: multi-scan (*CrysAlis RED*; Oxford Diffraction, 2005)	*k* = −18→18
*T*_min_ = 0.905, *T*_max_ = 0.964	*l* = −14→14
16118 measured reflections	

### Refinement

**Table:**

Refinement on *F*^2^	Secondary atom site location: difference Fourier map
Least-squares matrix: full	Hydrogen site location: difference Fourier map
*R*[*F*^2^ > 2σ(*F*^2^)] = 0.030	All H-atom parameters refined
*wR*(*F*^2^) = 0.082	*w* = 1/[σ^2^(*F*_o_^2^) + (0.0425*P*)^2^ + 0.2189*P*] where *P* = (*F*_o_^2^ + 2*F*_c_^2^)/3
*S* = 1.09	(Δ/σ)_max_ = 0.001
2969 reflections	Δρ_max_ = 0.32 e Å^−^^3^
217 parameters	Δρ_min_ = −0.20 e Å^−^^3^
0 restraints	Extinction correction: *SHELXL97* (Sheldrick, 2008), Fc^*^=kFc[1+0.001xFc^2^λ^3^/sin(2θ)]^-1/4^
Primary atom site location: structure-invariant direct methods	Extinction coefficient: 0.030 (3)

### Special details

**Table:**

Geometry. All e.s.d.'s (except the e.s.d. in the dihedral angle between two l.s. planes) are estimated using the full covariance matrix. The cell e.s.d.'s are taken into account individually in the estimation of e.s.d.'s in distances, angles and torsion angles; correlations between e.s.d.'s in cell parameters are only used when they are defined by crystal symmetry. An approximate (isotropic) treatment of cell e.s.d.'s is used for estimating e.s.d.'s involving l.s. planes.
Refinement. Refinement of *F*^2^ against ALL reflections. The weighted *R*-factor *wR* and goodness of fit *S* are based on *F*^2^, conventional *R*-factors *R* are based on *F*, with *F* set to zero for negative *F*^2^. The threshold expression of *F*^2^ > σ(*F*^2^) is used only for calculating *R*-factors(gt) *etc*. and is not relevant to the choice of reflections for refinement. *R*-factors based on *F*^2^ are statistically about twice as large as those based on *F*, and *R*- factors based on ALL data will be even larger.

### Fractional atomic coordinates and isotropic or equivalent isotropic displacement parameters (Å^2^)

**Table:**

	*x*	*y*	*z*	*U*_iso_*/*U*_eq_	
F1	0.91559 (8)	0.19749 (4)	0.55376 (5)	0.02345 (16)	
F2	0.72933 (9)	−0.06640 (5)	0.31548 (6)	0.02896 (18)	
O1	0.93207 (10)	0.03767 (6)	0.12362 (7)	0.02301 (19)	
O2	0.79654 (11)	0.07674 (6)	−0.07273 (7)	0.02281 (18)	
O3	0.88984 (10)	0.10342 (5)	0.33861 (6)	0.01920 (17)	
C1	0.63372 (13)	0.13060 (7)	0.08786 (9)	0.0169 (2)	
C2	0.46817 (14)	0.12551 (7)	0.01011 (10)	0.0212 (2)	
C3	0.31535 (15)	0.16368 (8)	0.04040 (11)	0.0264 (2)	
C4	0.32593 (15)	0.20931 (8)	0.14881 (12)	0.0270 (3)	
C5	0.48874 (15)	0.21687 (7)	0.22700 (10)	0.0228 (2)	
C6	0.64210 (13)	0.17778 (7)	0.19835 (9)	0.0172 (2)	
C7	0.81506 (14)	0.19166 (7)	0.28507 (9)	0.0193 (2)	
C8	0.81871 (13)	0.06741 (7)	0.43106 (9)	0.0162 (2)	
C9	0.83455 (13)	0.11215 (7)	0.54123 (9)	0.0176 (2)	
C10	0.77635 (15)	0.07254 (8)	0.63736 (10)	0.0216 (2)	
C11	0.70321 (15)	−0.01698 (8)	0.62442 (10)	0.0236 (2)	
C12	0.68763 (14)	−0.06530 (8)	0.51687 (10)	0.0230 (2)	
C13	0.74362 (14)	−0.02158 (7)	0.42232 (9)	0.0196 (2)	
B1	0.79588 (15)	0.08049 (8)	0.04625 (10)	0.0180 (2)	
H1O	0.921 (2)	0.0424 (11)	0.1939 (15)	0.039 (4)*	
H2O	0.877 (2)	0.0400 (12)	−0.0883 (14)	0.045 (4)*	
H2	0.4580 (17)	0.0929 (9)	−0.0654 (12)	0.024 (3)*	
H3	0.202 (2)	0.1592 (10)	−0.0139 (13)	0.035 (4)*	
H4	0.220 (2)	0.2362 (10)	0.1720 (13)	0.037 (4)*	
H5	0.4967 (18)	0.2490 (10)	0.3020 (12)	0.028 (3)*	
H7A	0.7984 (16)	0.2351 (9)	0.3485 (11)	0.019 (3)*	
H7B	0.9085 (16)	0.2154 (9)	0.2457 (11)	0.019 (3)*	
H10	0.7881 (19)	0.1076 (10)	0.7123 (13)	0.036 (4)*	
H11	0.6634 (19)	−0.0452 (10)	0.6898 (13)	0.032 (4)*	
H12	0.6353 (19)	−0.1267 (11)	0.5073 (12)	0.036 (4)*	

### Atomic displacement parameters (Å^2^)

**Table:**

	*U*^11^	*U*^22^	*U*^33^	*U*^12^	*U*^13^	*U*^23^
F1	0.0302 (3)	0.0207 (3)	0.0189 (3)	−0.0036 (2)	0.0035 (2)	−0.0040 (2)
F2	0.0401 (4)	0.0230 (3)	0.0228 (4)	−0.0024 (3)	0.0038 (3)	−0.0084 (3)
O1	0.0251 (4)	0.0324 (4)	0.0124 (4)	0.0089 (3)	0.0059 (3)	−0.0009 (3)
O2	0.0280 (4)	0.0264 (4)	0.0147 (4)	0.0068 (3)	0.0059 (3)	−0.0001 (3)
O3	0.0220 (4)	0.0218 (4)	0.0149 (4)	0.0044 (3)	0.0063 (3)	0.0009 (3)
C1	0.0195 (5)	0.0137 (5)	0.0180 (5)	0.0000 (4)	0.0049 (4)	0.0029 (4)
C2	0.0239 (5)	0.0163 (5)	0.0224 (5)	−0.0006 (4)	0.0019 (4)	0.0028 (4)
C3	0.0194 (5)	0.0209 (5)	0.0371 (6)	0.0005 (4)	0.0010 (5)	0.0076 (5)
C4	0.0231 (6)	0.0204 (5)	0.0402 (7)	0.0053 (4)	0.0131 (5)	0.0076 (5)
C5	0.0295 (6)	0.0165 (5)	0.0253 (6)	0.0040 (4)	0.0129 (5)	0.0031 (4)
C6	0.0217 (5)	0.0125 (4)	0.0187 (5)	0.0004 (4)	0.0071 (4)	0.0034 (4)
C7	0.0263 (5)	0.0167 (5)	0.0155 (5)	−0.0008 (4)	0.0056 (4)	0.0005 (4)
C8	0.0151 (4)	0.0197 (5)	0.0139 (5)	0.0048 (4)	0.0030 (3)	0.0015 (4)
C9	0.0175 (5)	0.0170 (5)	0.0173 (5)	0.0023 (4)	0.0006 (4)	−0.0006 (4)
C10	0.0253 (5)	0.0246 (5)	0.0147 (5)	0.0066 (4)	0.0034 (4)	0.0014 (4)
C11	0.0234 (5)	0.0263 (6)	0.0217 (5)	0.0060 (4)	0.0059 (4)	0.0092 (4)
C12	0.0216 (5)	0.0188 (5)	0.0277 (6)	0.0019 (4)	0.0028 (4)	0.0039 (4)
C13	0.0203 (5)	0.0194 (5)	0.0180 (5)	0.0041 (4)	0.0010 (4)	−0.0034 (4)
B1	0.0206 (5)	0.0175 (5)	0.0162 (5)	−0.0008 (4)	0.0046 (4)	−0.0011 (4)

### Geometric parameters (Å, °)

**Table:**

F1—C9	1.3589 (12)	C4—H4	0.978 (15)
F2—C13	1.3566 (12)	C5—C6	1.3948 (15)
O1—B1	1.3708 (14)	C5—H5	0.959 (14)
O1—H1O	0.821 (16)	C6—C7	1.5041 (15)
O2—B1	1.3536 (14)	C7—H7A	0.976 (13)
O2—H2O	0.853 (17)	C7—H7B	0.976 (12)
O3—C8	1.3727 (12)	C8—C13	1.3865 (15)
O3—C7	1.4630 (12)	C8—C9	1.3884 (14)
C1—C2	1.4010 (15)	C9—C10	1.3776 (15)
C1—C6	1.4139 (14)	C10—C11	1.3880 (16)
C1—B1	1.5825 (15)	C10—H10	0.976 (15)
C2—C3	1.3934 (16)	C11—C12	1.3868 (16)
C2—H2	0.965 (13)	C11—H11	0.946 (15)
C3—C4	1.3806 (18)	C12—C13	1.3795 (16)
C3—H3	0.966 (15)	C12—H12	0.959 (15)
C4—C5	1.3891 (17)		
			
B1—O1—H1O	112.4 (11)	O3—C7—H7B	103.0 (7)
B1—O2—H2O	112.0 (10)	C6—C7—H7B	112.2 (7)
C8—O3—C7	117.18 (7)	H7A—C7—H7B	109.4 (10)
C2—C1—C6	117.67 (9)	O3—C8—C13	120.47 (9)
C2—C1—B1	117.17 (9)	O3—C8—C9	122.68 (9)
C6—C1—B1	125.14 (9)	C13—C8—C9	116.49 (9)
C3—C2—C1	121.77 (10)	F1—C9—C10	119.62 (9)
C3—C2—H2	118.7 (8)	F1—C9—C8	117.57 (9)
C1—C2—H2	119.5 (8)	C10—C9—C8	122.79 (10)
C4—C3—C2	119.76 (11)	C9—C10—C11	118.53 (10)
C4—C3—H3	119.4 (9)	C9—C10—H10	119.5 (9)
C2—C3—H3	120.9 (9)	C11—C10—H10	121.9 (9)
C3—C4—C5	119.83 (10)	C12—C11—C10	120.83 (10)
C3—C4—H4	121.1 (9)	C12—C11—H11	119.7 (9)
C5—C4—H4	119.1 (9)	C10—C11—H11	119.5 (8)
C4—C5—C6	120.90 (10)	C13—C12—C11	118.39 (10)
C4—C5—H5	120.0 (8)	C13—C12—H12	120.6 (8)
C6—C5—H5	119.1 (8)	C11—C12—H12	120.9 (8)
C5—C6—C1	120.05 (10)	F2—C13—C12	120.07 (9)
C5—C6—C7	118.09 (9)	F2—C13—C8	117.00 (9)
C1—C6—C7	121.78 (9)	C12—C13—C8	122.92 (10)
O3—C7—C6	112.62 (8)	O2—B1—O1	118.25 (9)
O3—C7—H7A	109.5 (7)	O2—B1—C1	118.09 (9)
C6—C7—H7A	110.0 (7)	O1—B1—C1	123.62 (9)
			
C6—C1—C2—C3	−1.23 (15)	C13—C8—C9—F1	176.87 (8)
B1—C1—C2—C3	177.16 (10)	O3—C8—C9—C10	−174.44 (9)
C1—C2—C3—C4	1.10 (16)	C13—C8—C9—C10	−1.33 (15)
C2—C3—C4—C5	−0.02 (16)	F1—C9—C10—C11	−176.36 (9)
C3—C4—C5—C6	−0.90 (16)	C8—C9—C10—C11	1.80 (16)
C4—C5—C6—C1	0.74 (15)	C9—C10—C11—C12	−0.50 (16)
C4—C5—C6—C7	177.80 (9)	C10—C11—C12—C13	−1.17 (16)
C2—C1—C6—C5	0.31 (14)	C11—C12—C13—F2	−179.55 (9)
B1—C1—C6—C5	−177.94 (10)	C11—C12—C13—C8	1.67 (16)
C2—C1—C6—C7	−176.63 (9)	O3—C8—C13—F2	−6.00 (14)
B1—C1—C6—C7	5.11 (15)	C9—C8—C13—F2	−179.27 (8)
C8—O3—C7—C6	−79.20 (11)	O3—C8—C13—C12	172.82 (9)
C5—C6—C7—O3	115.02 (10)	C9—C8—C13—C12	−0.46 (15)
C1—C6—C7—O3	−67.98 (12)	C2—C1—B1—O2	31.58 (14)
C7—O3—C8—C13	121.32 (10)	C6—C1—B1—O2	−150.16 (10)
C7—O3—C8—C9	−65.84 (12)	C2—C1—B1—O1	−145.87 (10)
O3—C8—C9—F1	3.76 (14)	C6—C1—B1—O1	32.39 (16)

### Hydrogen-bond geometry (Å, °)

**Table:**

*D*—H···*A*	*D*—H	H···*A*	*D*···*A*	*D*—H···*A*
O1—H1O···O3	0.821 (16)	1.915 (16)	2.6926 (11)	157.7 (15)
O2—H2O···O1^i^	0.853 (17)	1.937 (17)	2.7889 (11)	176.9 (16)

Symmetry codes: (i) −*x*+2, −*y*, −*z*.
